# Lea’s Series of Medical Epitomes

**Published:** 1904-10

**Authors:** 


### Lea's Series of Medical Epitomes.

Magee & Johnson's Epitome of Surgery. A Manual for Students and Practitioners. By M. D'Arcy Magee, A.M., M.D., Demonstrator of Surgery and Lecturer on Minor Surgery, and Wallace Johnson, Ph.D., M.D., Demonstrator of Pathology and Bacteriology in Georgetown University Medical School, Washington, D. C. In one 12mo volume of 295 pages, with 129 engravings. Price, cloth, $1, net. Philadelphia and New York: Lea Bros. & Co. 1904.

